# The Hurdle of Access to Emerging Therapies and Potential Solutions in the Management of Dyslipidemias

**DOI:** 10.3390/jcm13144160

**Published:** 2024-07-16

**Authors:** Brett S. Mansfield, Farzahna Mohamed, Miriam Larouche, Frederick J. Raal

**Affiliations:** 1 Department of Internal Medicine, University of the Witwatersrand, Johannesburg 2193, South Africa; brett.mansfield@wits.ac.za (B.S.M.); farzahna.mohamed@wits.ac.za (F.M.); 2 Carbohydrate & Lipid Metabolism Research Unit, University of the Witwatersrand, Johannesburg 2193, South Africa; 3 Département de Médecine, Université de Montréal and ECOGENE-21, Montreal, QC H3T 1J4, Canada; miriam.larouche@ecogene21.org

**Keywords:** lipid-lowering therapies, dyslipidemia, access, barriers, ASCVD, public health, telemedicine

## Abstract

This review explores the many barriers to accessing lipid-lowering therapies (LLTs) for the prevention and management of atherosclerotic cardiovascular disease (ASCVD). Geographical, knowledge, and regulatory barriers significantly impede access to LLTs, exacerbating disparities in healthcare infrastructure and affordability. We highlight the importance of policy reforms, including pricing regulations and reimbursement policies, for enhancing affordability and streamlining regulatory processes. Innovative funding models, such as value-based pricing and outcome-based payment arrangements, have been recommended to make novel LLTs more accessible. Public health interventions, including community-based programs and telemedicine, can be utilized to reach underserved populations and improve medication adherence. Education and advocacy initiatives led by patient advocacy groups and healthcare providers play a crucial role in raising awareness and empowering patients. Despite the barriers to access, novel LLTs present a big opportunity to reduce the burden of ASCVD, emphasizing the need for collaborative efforts among policymakers, healthcare providers, industry stakeholders, and patient advocacy groups to address these barriers to improve access to LLTs globally.

## 1. Introduction

Atherosclerotic cardiovascular disease (ASCVD) remains the leading cause of morbidity and mortality worldwide [1]. More than 16 million people died from coronary artery disease or stroke in 2021 [1].

With advances in therapeutics, deaths due to ASCVD have steadily declined in most high-income countries (HICs), apart from the US which has seen a resurgence in cardiovascular mortality over the past decade [1,2]. Indeed, more people died from cardiovascular disease in the US in 2020 than in any other year since 2003 [2]. Possible reasons for this resurgence include a rising prevalence of obesity, metabolic syndrome, diabetes, and hypertension [2]. The impact of the coronavirus 2019 (COVID-19) pandemic, too, has led to excess cardiovascular mortality [2].

In addition, cardiovascular mortality in low- and middle-income countries (LMICs) remains unacceptably high, being nearly twice that of HICs [1]. Indeed, 80% of all deaths due to cardiovascular disease (CVD) occur in LMICs, with the prevalence of ASCVD and the burden of risk factors expected to rise exponentially [3,4]. The projected economic burden of risk factors and the consequences of ASCVD is expected to triple over the next 30 years [5].

Dyslipidemia, in particular, elevated low-density lipoprotein cholesterol (LDL-C), is one of the major modifiable risk factors that contribute to ASCVD [6]. This review will address therapies aimed at lowering LDL-C. Statins have been the cornerstone of lipid-lowering therapy (LLT) and have been listed on the World Health Organization (WHO) Model of Essential Medicines List (EML) since 2007 [7]. Statins are recommended for ASCVD prevention globally but remain underutilized and underdosed, especially in LMICs [4,7]. In low-income countries (LICs), only 19.8% of patients receive medicines for secondary prevention of ASCVD, compared to 54.9% in upper-middle-income countries (UMICs) [8,9].

Treatment guidelines continue to recommend lower LDL-C targets, particularly for subjects with established ASCVD, resulting in the need for additional non-statin LLTs [10]. Many patients are undertreated and do not reach current guideline-directed LDL-C targets [11,12]. Furthermore, up to 30% of patients report statin-related side effects [13,14]. Although the nocebo effect of statins is well established, poor perceptions surrounding statin use contribute to poor adherence [15,16,17]. As such, there is a need for non-statin LLTs to bridge this treatment gap.

The United Nations has set a Sustainable Development Goal to reduce premature mortality from non-communicable diseases (NCDs) by one-third by 2030 [3,18,19]. If this goal is to be realized, then improved access to essential medicines, including novel and emerging lipid-lowering therapies (LLTs), will need to be prioritized.

Significant differences exist in access to medications in different countries and regions around the world. While it is not possible to identify the specific barriers for all countries, this review will highlight just some of the difficulties that exist in providing novel therapies to the patients who need them.

## 2. Novel Lipid-Lowering Therapies

Over the past few decades, a number of new drugs have been approved for LDL-C reduction.

Evolocumab (Repatha) and alirocumab (Praluent), monoclonal antibody therapies directed against proprotein convertase subtilisin/kexin type 9 (PCSK9), are administered every 2–4 weeks as subcutaneous injections. They demonstrated a 50–60% reduction in LDL-C and reduced ASCVD mortality in the FOURIER and ODYSSEY OUTCOMES trials, respectively [20,21]. PCSK9 inhibitors are recommended for patients with ASCVD or at high risk for ASCVD who cannot achieve adequate lowering of LDL-C with maximally tolerated statin therapy or for those who are statin-intolerant [22]. Likely because of cost, the uptake of PCSK9 inhibitor therapy has been poor, with most users being in the US [11,12,23].

Tafolecimab (Sintbilo), a humanized monoclonal antibody targeting PCSK9, received approval in China in August 2023 [24]. Dosed by monthly subcutaneous injection, tafolecimab led to a 57–70% reduction in LDL-C in patients with and without FH [24,25,26]. As many as 10% of participants developed anti-drug antibodies and 1% developed neutralizing antibodies during a 48-week trial period [26].

Inclisiran (Leqvio or Sybrava) is a small interfering RNA (siRNA) therapy directed against the hepatic synthesis of PCSK9 and leads to an approximately 50% reduction in LDL-C [27,28]. Over and above the traditional indications for PCSK9 inhibitor therapy, inclisiran can be considered in individuals with poor adherence to or adverse effects from the PCSK9 monoclonal antibodies [22]. A significant benefit of inclisiran over evolocumab and alirocumab is its 6-monthly dosing schedule.

The widespread use of inclisiran has been significantly hampered in the UK, with less than 5000 prescriptions for the drug occurring over 18 months [29]. This number falls grossly short of the National Health Services (NHS) target of 300,000 patients by October 2024 [29]. Major factors limiting the prescribing of inclisiran include uncertainty around costs, drug safety, and the bureaucratic workload of prescribing the drug [30].

Bempedoic acid is an orally administered drug that inhibits adenosine triphosphate (ATP)-citrate lyase, an enzyme located upstream of 3-hydroxy-3-methylglutaryl coenzyme A (HMG Co-A) reductase, the rate-limiting enzyme targeted by statins [31]. Bempedoic acid leads to approximately a 24% reduction in LDL-C in statin-intolerant individuals and has a low rate of myalgias [32]. When added to maximally tolerated statin therapy, bempedoic acid reduces LDL-C by between 16 and 18% [33,34]. Moreover, the CLEAR Outcomes trial demonstrated a reduction in the incidence of ASCVD in individuals who were intolerant of statins [35].

While the monthly costs of bempedoic acid (registered as Nexletol in the USA and Nilemdo in the EU) and its combination with ezetimibe (Nexlizet in the USA and Nustendi in the EU) are cheaper than PCSK9 inhibitors, because of the smaller percentage reduction in LDL-C, they are more costly per mg/dL reduction in LDL-C achieved.

Evinacumab (Evkeeza) is a monoclonal antibody that inhibits angiopoietin-like protein 3 (ANGPTL3), lowering LDL-C by LDL-receptor-independent means. Remarkably, reductions in LDL-C levels by approximately 50% in addition to maximally tolerated LLT were seen in the ELIPSE-HoFH trial [36]. The drug has been approved for the treatment of homozygous familial hypercholesterolemia (HoFH) from the age of 5 years [36,37].

Newer therapies at various stages in the research and development pipeline are likely to become available in the future.

- Lerodalcibep, a recombinant fusion protein of a PCSK9-binding domain (adnectin) with human albumin, reduced LDL-C by over 50% with monthly subcutaneous injections when administered to heterozygous FH patients over a 24-week study period [38].
- Oral PCSK9 inhibitors have shown similar reductions to the injectable PCSK9 inhibitors [39,40]. NNC0385-0434 was withdrawn for commercial reasons, while MK-0616 is currently in phase 3 clinical trials.
- The cholesterol ester transfer protein (CETP) inhibitors as high-density lipoprotein cholesterol (HDL-C)-raising therapies did not reduce atherosclerosis or cardiovascular events [41,42,43]. However, obicetrapib, a novel CETP inhibitor, in addition to raising HDL-C, has demonstrated a 45% reduction in median LDL-C in a randomized phase 2 study and a 63% reduction in combination with ezetimibe [44,45].
- Zodasiran is an siRNA molecule that disrupts the expression of *ANGPTL3* [46]. The ARCHES-2 trial showed that, at varying doses, zodasiran was effective in patients with mixed hyperlipidemia, leading to a 51–63% reduction in triglycerides (TGs) and 14–20% reduction in LDL-C [46].
- Solbinsiran, a GalNAC-conjugated siRNA against ANGPTL3, produced a dose-dependent reduction in TG, non-HDL-C, and apolipoprotein B in individuals with mixed hyperlipidemia [47].
- CRISPR/Cas9-based gene editing therapies directed against the *PCSK9* and *ANGPTL3* genes are entering human trials given the safety shown in preclinical models [48,49,50]. These are considered good targets given the lifelong absence of any health-related consequences in individuals with naturally occurring loss-of-function *PCSK9* and *ANGPTL3* mutations.
- Vaccination strategies inducing a host immune response against circulating PCSK9 or ANGPTL3 remain within the preclinical domain but have shown promising results in non-human primates and other animal models [51,52,53,54].

It remains to be seen whether these new therapies (displayed in Table 1) will reduce cardiovascular events, and further studies are awaited. Some are indeed likely to obtain regulatory approval in the future and will enter the market alongside the currently available lipid-lowering therapies. However, the question of how accessible these therapies will be to the general population is of concern, especially considering the global underutilization of many of the currently available lipid-lowering therapies, particularly within LMICs [11,12,55].

## 3. Barriers to Accessing Medicines

The concept of access to medicines was defined by Pechansky and Thomas in 1981 and encompasses five key dimensions: availability, accessibility, affordability, acceptability, and quality [56].

Availability ensures that medications are supplied sufficiently, while affordability focuses on making them economically accessible to all patients. Accessibility addresses the physical reachability and obtainability of medicines, and acceptability considers the cultural and personal preferences of patients. Finally, quality refers to safe and effective medicines that are of a consistently high standard [56,57]. Collectively, these dimensions form a comprehensive framework for evaluating and improving access to essential medicines globally.

The World Health Organization (WHO) defines access as “having medicines continuously available and affordable within one hour’s walk from home” [8]. The United Nations’ Sustainable Development Goal 3.8 proclaims “…access to safe, effective, quality and affordable essential medicines and vaccines for all” [58].

Access to therapies will be considered in terms of economic, geographical, regulatory, and knowledge barriers.

### 3.1. Economic Barriers: The Availability and Affordability of Medicines

Availability has been defined by the WHO as the percentage of medicine outlets or facilities having a particular medication in stock at any one time [57,59,60]. A threshold of 80% is the target set by the WHO to declare the availability of essential affordable medicines within a particular region [57,59,60]. This reflects an understanding that while 100% availability may be ideal, it is often impractical due to logistical, economic, and systemic challenges that health systems may face. This is particularly true of LMIC, and thus, 80% serves as a realistic and impactful target.

The WHO determines affordability according to the number of days’ wages needed to buy a one-month supply of medicines for chronic conditions based on the daily wage of the lowest-paid unskilled government worker. Medicines are considered unaffordable if the total cost exceeds 20% of the household income [57,59].

Economic barriers significantly impact the availability and affordability of LLTs across different income regions. Despite the clinical efficacy of lipid-lowering therapies, the financial implications often hinder their widespread adoption. High-intensity statins and ezetimibe are generally available, although significant treatment gaps remain [3,12,55].

The World Heart Federation survey drew respondents from 38 countries, with most being from the US and Europe and most working in an urban setting [3]. In LICs, 40% reported that fixed-dose statin/ezetimibe combinations were not available, and 60% noted that PCSK9 inhibitors were not available [3]. These availability issues are less pronounced in UMICs and HICs, highlighting the disparities in access [3].

Generic drugs are more affordable than patented drugs as they are usually priced more closely to the cost of production [61]. Significant disparities exist both within and between countries in terms of the availability of generic medications. Up to 20% of generic medicines are available in the public sector, whereas 60% can be found in the private sector in LMICs [60]. Poor availability of medicines in the public sector often forces patients to seek more expensive options from private facilities [60].

In patients surveyed across 21 countries (HICs, UMICs, LMICs and LICs), the availability of CV medicines was found to be 50% for antihypertensives, 62.8% for anti-platelets, and 87.2% for statins [55,59]. Importantly, 29.4% of providers who were surveyed reported that while the drugs were available but not affordable, and 25.7% lacked access to all three drugs [59]. Only 51% of participants were using a lipid-lowering agent, with 80% of participants remaining with an LDL-C above the target of 100 mg/dL [62]. CV medicines are generally unaffordable in most countries, particularly in LICs, as 75% of households in LICs and 24% in MICs could not afford two antihypertensive drugs and a statin.

Post-manufacture costs, including duties, taxes, markups, and additional charges, further increase the financial burden on patients [57,59]. In LMICs, inadequate public financing limits the availability of essential CV medicines, despite the implementation of social health insurance in some areas [3,59]. Patients without insurance who pay out of pocket often find medicines unaffordable. The affordability of combination therapy for secondary prevention of CVD is also problematic, with 33% of households in LMICs and 60% in LICs being unable to afford such treatment [57,59]. Increased copayments for medicines have been shown to reduce usage, though this evidence primarily comes from HICs [57].

Significant variability has been seen in the pricing of PCSK9 inhibitors worldwide, ranging from USD 127 per standard unit (SU) in Korea to USD 949 per SU in Argentina [23].

The affordability of medicines is often compared to the international reference price. Patented medication manufacturer prices in the US are typically 20–40% more compared to other HICs, whereas generics are cheaper in the US [63]. Government-procured generics are priced 1.5 to 3 times higher than the reference prices, while the same generics cost patients approximately 15 times the reference price in the public sector and 30 times in the private sector [57]. In the public sector, a one-month supply of a generic CVD medicine costs an average of 2 days’ wages, whereas an original brand costs 8.3 days’ wages for the lowest-paid government worker [57,59]. Procurement prices in LMICs are significantly higher than international reference prices, averaging 17 times higher for brand medicines and 4.5 times higher for generics [59]. Patient prices are even more inflated, being 11.2 times the international reference price for generics in the public sector [59]. The disparities in access to CV medicine between the private and public sectors, and across different countries and income levels, are significant. In the private sector, these medicines are more readily available but often less affordable, highlighting the crucial role both sectors play in ensuring access to CV treatments [59].

The high cost of novel LLTs and the increasing burden of NCDs challenge the feasibility and sustainability of universal health coverage. This contributes to the increasing burden of CVD in LMICs, where more than three-quarters of CVD-related deaths occur [59].

### 3.2. Geographical Barriers: Disparities in Accessibility Based on Location and Healthcare Infrastructure

Geographical barriers affect accessibility based on location and the quality of healthcare infrastructure. Structural barriers, such as limited national funding, slow inclusion of CV medicines in EMLs, and supply chain inefficiencies, exacerbate the problem, making guideline-based treatment challenging [57,59,64]. Studies have shown that the inadequate presence of healthcare workers and long distances to health facilities are significant challenges to accessing medicines and healthcare in LMICs [59].

In sub-Saharan Africa, patients often travel long distances to access health facilities. Indeed, up to 35% of patients with chronic diseases in LMICs travel more than 15 min to visit health facilities [59]. Accessibility is crucial, as patients must be able to obtain medicines even if they are affordable. Inefficient transportation systems, infrastructural inadequacies, a lack of accountability, and a low density of healthcare workers are major barriers to accessing CV medicines [57,59]. High rates of absenteeism among public-sector health workers can further hinder this access [57,59].

In LMICs, geographical location greatly affects access to NCD medicines, with those living in the capital having better access than rural residents, and long travel times to health facilities significantly reducing overall access [57]. Significant disparities exist between the public and private sectors, urban and rural areas, and various income levels, with CV medicines being more available but less affordable in the private sector, highlighting the need for policy measures to ensure access across all regions, especially in low-income economies facing high CV morbidity and mortality [59].

### 3.3. Knowledge Barriers: Limited Awareness and Acceptability among Healthcare Providers and Patients about New Treatment Options

Low acceptability among patients and healthcare workers, compounded by physicians’ preferences for non-EML brands and complex treatment regimens, leads to poor patient adherence to medications and non-compliance with local guidelines [57,59]. This results in low treatment rates and medication adherence, despite the availability of medicines. Adherence to daily LDL-C-lowering medications is hindered by patient factors like risk perception and health literacy [65].

Non-adherence by both healthcare providers and patients further reduces the acceptability of treatments. Patient beliefs about the necessity and potential side effects of medication further contribute to non-adherence [57]. The World Heart Federation survey highlighted further roadblocks and potential solutions in ASCVD management [3]. The survey found significant disparities in clinician comfort with prescribing high-potency statins, especially in LMICs, where 60% of respondents were uncomfortable with such prescriptions compared to 35% in HICs [3].

Patient perceptions play a crucial role, with many patients in LICs finding it challenging to understand the risks associated with ASCVD and the benefits of cholesterol-lowering medications, leading to low treatment acceptance and adherence [3]. Healthcare systems often focus on treating diseases in secondary and tertiary care settings rather than on primary care and health preservation, presenting a major barrier to ASCVD prevention [3]. There is also a gap between the awareness of what combination therapies to use and their correct application, indicating the need for improved education and training among healthcare providers [3]. Simplifying prescription access through electronic solutions could help, but low awareness and acceptance of new treatments like PCSK9 inhibitors and siRNA-based therapies among providers and patients remain significant barriers [65].

Overall, the acceptability of CV medicines is influenced by a range of factors, including provider and patient perceptions, the complexity of treatment regimens, and the availability of high-quality medicines. Addressing these issues is crucial for improving CV disease outcomes, particularly in LMICs. Additionally, patent laws and systemic deficiencies contribute to the burden of counterfeit drugs in LMICs [59].

### 3.4. Regulatory Barriers: Delays in Approval Processes, Access Restrictions, and Quality Assurance

The prevalence of substandard and counterfeit medicines presents a major challenge, particularly in Africa, where regulatory oversight is often insufficient, with a recent study indicating that 16.3% of CV medicines in Africa are of low quality, making them one of the most commonly reported substandard medicine classes in the WHO monitoring system [59]. Concerns about medicine quality are global, leading to interventions like regular quality testing by large procurement agencies to ensure a consistent supply of quality products from manufacturers or distributors [57].

The substantial financial burden incurred by novel LLTs prompts insurers to implement stringent cost-containment strategies such as prior authorization (PA) requirements and step therapy [66]. The PA process, involving extensive paperwork and data collection, causes delays and discourages access, while step therapy mandates that patients try and fail on less expensive medications before being approved for novel agents like PCSK9 inhibitors, further delaying access to effective treatment [66]. These regulatory hurdles are compounded by a burdensome appeals process, which diverts healthcare providers’ time and resources away from patient care, sometimes even imposing financial burdens on physicians through filing fees for appeals [66].

Over a period of 3 years, 91% of initial claims for PSCK9 inhibitors through commercial insurers were rejected. Following an appeals process, this figure declined to 53% [66]. But, importantly, 74% of patients who were not granted access to PCSK9 inhibitors did not commence alternative LLT [66]. In people presumed to have FH who were not meeting the recommended LDL-C targets, more than 60% of applications for PCSK9 inhibitors were rejected [67].

Meeting rigorous criteria to access therapies PCSK9 is time-consuming [68]. The bureaucracy related to these regulatory difficulties can inhibit healthcare provider prescribing [29,30]. The delays and denials can have serious implications for patient outcomes, particularly for those at high risk for cardiovascular events. Timely access to PCSK9 inhibitors is crucial for optimal lipid management and reducing ASCVD risk [66].

Inconsistencies in the approval processes further complicate access, with similar clinical histories receiving different decisions, undermining trust in the system. Additionally, the administrative burden of managing these processes imposes significant costs on medical practices, straining healthcare resources and disrupting the essential patient–clinician relationship [66].

The biannual update of the WHO Model List of Essential Medicines serves as a guide for countries to create their own prioritized medication lists according to local health needs, but applying comparative cost-effectiveness on a global scale is difficult [57]. A study conducted in sub-Saharan Africa revealed that 40% of countries had not revised their EMLs in the past five years [57]. LICs generally include CVD medicines less frequently than HICs, and only about half of countries include at least one medicine from each of the four secondary prevention therapeutic groups (aspirin, beta-blocker, ACE inhibitor, and statin) on their EMLs, with statin inclusion rates of 43%, 75%, and 69% for LICs, LMICs, and HICs, respectively [57]. The availability of generic medicines for acute conditions is generally higher than that for chronic conditions in both public and private sectors [57].

HICs have stringent regulatory frameworks (e.g., FDA and EMA) that thoroughly evaluate the safety and efficacy of novel LLTs. LMICs may possess regulatory authorities that are underfunded with inadequate expertise, which leads to less rigorous or inconsistently enforced standards that result in variations in drug quality and availability. HICs can also benefit from quicker access to novel therapies with fast-track regulatory approval programs.

### 3.5. Systemic Racism as a Barrier to Access

Structural inequalities, including systemic racism, play a significant role in shaping healthcare access and outcomes [69]. Racism within healthcare systems can manifest in various forms, including unequal treatment, bias in diagnosis and treatment decisions, and disparities in access to quality care based on race or ethnicity [69,70,71]. Systemic racism contributes to socioeconomic disparities, lack of health insurance coverage, and limited access to healthcare facilities, exacerbating the challenges in obtaining necessary treatments [69]. Racial disparities are seen throughout the field of medicine and, no less, in the access to LLTs [71,72].

## 4. Barriers and Their Budget Impact on CV Outcomes

A major barrier to adequate LDL-C lowering in LICs is the lack of registration for these therapies, as 75% of respondents in LICs cited the absence of registration for fixed-dose statin/ezetimibe combinations and PCSK9 inhibitors [3]. While LMICs also face registration challenges, they are less severe, with only 10% of UMIC respondents reporting issues with PCSK9 inhibitors [3].

Regarding prescription practices, none of the four treatments (high-intensity statins, ezetimibe, fixed-dose combinations, or PCSK9 inhibitors) are universally prescribed freely in the public sector in LICs [3]. Only 50% of respondents in LICs noted that high-intensity statins could be freely prescribed, whereas approximately 75% reported that ezetimibe, fixed-dose combinations, and PCSK9 inhibitors could not be freely prescribed [3]. In contrast, HICs typically allow most LLTs, apart from PCSK9 inhibitors, to be prescribed freely in the public sector.

Raised LDL-C in young adults is linked to later life ASCVD, yet most do not receive LLT. A study, using the U.S. National Health and Nutrition Examination Survey (NHANES) database, found that initiating statin treatment for young adults with LDL-C ≥ 130 mg/dL is highly cost-effective in men (USD 31,000/quality-adjusted life-year [QALY]) and moderately cost-effective in women (USD 106,000/QALY), with lifestyle interventions being less effective and more costly [73].

LLTs are most cost-effective in high-risk populations. The higher the risk, the greater the absolute risk reduction when the treatment is given, making the intervention more cost-effective [74]. Since men typically have higher baseline risk and higher rates of ASCVD events, lowering risk and preventing events lead to a more significant increase in QALYs for men as compared to women [74,75]. Furthermore, women generally have a longer life expectancy than men and, although this means they may benefit from more years of treatment, it also means costs are spread over a longer period of time.

Low/moderate-intensity statins combined with ezetimibe is the most cost-effective lipid-lowering strategy, especially when initiated at age 40, with an incremental cost-effectiveness ratio of GBP 11,107 per QALY gained [76]. PCSK9 inhibitors, alirocumab and evolocumab, face significant access barriers despite their FDA approval in 2015 for lowering LDL-C in patients with FH and ASCVD [66].

A budget impact study was conducted, using a 3-year model of introducing PCSK9 inhibitors to treat adults with heterozygous FH or established ASCVD requiring additional LDL-C lowering for a hypothetical US health plan with one million members [77]. The estimated costs over three years with the maximum PCSK9 inhibitor utilization of 1–5%, were low, with a total healthcare budget impact per patient per month of USD 3.62 in year 1, USD 7.22 in year 2, and USD 10.79 in year 3 [77]. These costs are sensitive to the model’s timeframe and the cost of PCSK9 inhibitors but remain modest compared to other specialty biologics. Drug cost rebates and discounts could further reduce the budget impact [77].

A cost analysis study focused on the community taxpayers’ perspective in South-Eastern Italy analyzed the costs per major cardiovascular event saved using data from randomized controlled trials [78]. Individual costs per saved adverse event ranged from EUR 0.12 to EUR 0.78. For every EUR 1 spent per inhabitant annually, 2–8.3 major adverse cardiovascular events could be avoided, demonstrating that the cost per event saved with PCSK9 inhibitors can translate into very small individual costs per year [78].

In Canada, it was estimated that 51.9% of patients with ASCVD would be eligible for PCSK9 inhibitors. Although the adoption of PCSK9 inhibitors was expected to reduce primary event rates by 1.8% after three years and save USD 44 million in events, the net budget impact over three years was USD 1.5 billion [79]. Targeting high-risk subgroups could further reduce these costs and lessen the overall budgetary impact, making the adoption of PCSK9 inhibitors more economically feasible [78,79].

Inclisiran, a small interfering RNA directed against PCSK9, was found to be the least cost-effective, not being viable in any subgroup at its current price [76]. Despite being effective, inclisiran was not cost-effective in any simulation, with its current price (GBP 3974.72) far exceeding the maximum cost-effective price (GBP 451 for men with LDL-C ≥ 200 mg/dL) [76]. This highlights the need for significant price reductions for inclisiran to be a viable option for primary prevention of coronary heart disease, especially considering the cost-effectiveness of statin-based strategies even with simulated non-adherence. Additionally, for secondary prevention, inclisiran would also require a substantial price reduction to be considered cost-effective compared to other PCSK9 inhibitors [76].

Cost is an even greater barrier for novel therapies such as evinacumab for the treatment of HoFH. Although clinical trials demonstrated significant LDL-C reductions, evinacumab is not cost-effective at its current price, with an Institute for Clinical and Economic Review (ICER) of USD 8,392,585 per QALY gained, far exceeding the USD 50,000 per QALY threshold. The drug costs about USD 460,839 per year per patient, highlighting the need for a significant price reduction [37]. Despite these challenges, evinacumab addresses an important unmet need, reducing morbidity and mortality in HoFH patients [80].

Despite the higher cost of novel LLTs, there is a significant benefit in their use for reducing CVEs. Acute coronary syndromes, coronary interventions, stroke, and cardiac arrest are more prevalent in patients with rejected or abandoned PCSK9 inhibitor prescriptions compared to those with paid PCSK9 inhibitor prescriptions [81]. Higher rejection rates were observed among women, racial minorities, and lower-income groups [81]. This is further reinforced by a sub-analysis of the Prospective Urban Rural Epidemiology (PURE) study, representative of different income regions, which showed that the lower availability and affordability of essential CVD medicines were associated with a higher risk of MACEs and mortality [55].

These studies highlight the critical need for improving access to these therapies to reduce CVEs globally and that, despite the initial costs, the long-term benefits of using LLTs, particularly in high-risk patients, justify their use. By preventing costly CVEs, these therapies can ultimately reduce the financial burden on healthcare systems, especially when strategic measures like targeted high-risk group treatments and cost-sharing initiatives are employed. Therefore, improving the availability and affordability of these medications should be prioritized as a cost-effective strategy to enhance CV health outcomes globally. Affordability was often lower in the private sector despite higher availability [59].

Further cost-effectiveness studies are required in a variety of contexts. Studies that evaluate lost productivity, work absenteeism, and long-term disability can also contribute to the understanding of both the economic and clinical impact of access or lack of access to LLT in different countries and regions.

## 5. Potential Solutions for Improving Access

### 5.1. Healthcare Policy Reforms

#### 5.1.1. Pricing Regulations

Access to medications is often limited by high costs that can neither be funded by individuals nor the countries in which they live. This is a concern for patients and health systems worldwide [82].

The WHO guideline on country pharmaceutical pricing policies refers to 10 pricing policies that countries can consider when managing medicine prices.

Government intervention in pharmaceutical pricing policies can make these therapies more affordable and thus more accessible to the broader population [82].

Value-based pricing (VBP), a strategy recommended by the WHO, is an approach that considers the value and benefits of a treatment when determining its price, thereby aligning the cost of a drug with its clinical benefits and overall value to the health system [82].

Implementing a VBP system presents several challenges due to the differences in healthcare systems as well as the differences in reimbursement found across countries and regions. VBP relies on clinical outcomes and real-world evidence to determine cost-effectiveness, but these data may be inconsistent, incomplete, or unavailable [83,84]. Ongoing evaluation of drug performance can be administratively burdensome and costly, but it can lead to drug prices changing over time as new evidence emerges [83,84]. The VBP model also requires buy-in from pharmaceutical companies, who may resist lower prices which could ultimately threaten the introduction of newer therapies onto the market [83,84].

Moreover, VBP may also lead to disparities in access. Wealthier countries have the resources to conduct comprehensive cost-effectiveness analyses and have different thresholds for determining cost-effectiveness in their healthcare systems [83,84].

External reference pricing (ERP), also known as international price referencing, is a model where drug prices are set based on the prices of the same drug in other countries [85,86,87,88]. ERP can be more effective in LMICs as it allows these countries to leverage lower prices from other countries [86,87]. ERP can be easier to implement, more effective for immediate price control, and more transparent compared to internal reference pricing (IRP) [86,87,88]. It provides a clear framework based on observed international prices, which helps when negotiating prices with pharmaceutical companies [89,90]. Canada uses ERP to align its drug prices with those in other developed countries. This has helped to keep drug prices relatively low while maintaining access to new therapies [89,90].

By contrast, IRP involves setting the price of a new drug relative to the prices of similar drugs within a country [85]. This model encourages competition, which drives prices down but may limit the entry of novel agents if they are priced too aggressively compared to existing therapies [82,85]. IRP can encourage the introduction of generic and biosimilar drugs into the market, as manufacturers try to offer more cost-effective alternatives to reference-priced drugs [88].

If reference prices are set too low, however, it could reduce the profitability of innovative drugs, which would discourage investment in high-risk, high-reward research and development [88].

Both ERP and IRP have strengths and weaknesses, and their effectiveness can vary based on the specific economic and healthcare contexts of different countries. Many countries adopt a hybrid approach, integrating aspects of both ERP and IRP to achieve a balance between access, affordability, and innovation [88].

Countries such as Canada, Germany, the UK, Australia, and China have implemented various policy regulations which have led to lower drug prices [90,91,92,93].

Health technology assessment (HTA) is a multidisciplinary process that evaluates the medical, social, economic, and ethical implications of using a health technology (drug, medical device, or procedure) [93]. HTA aims to provide evidence-based information about efficacy, safety, and cost-effectiveness to assist with decision making regarding the reimbursement and use of a therapy within health systems [93]. HTA aims to do this in a systematic, transparent, and unbiased manner [94]. Consequently, HTA can greatly influence the cost and reimbursement status of a medication and is, thus, an important tool for prioritizing healthcare spending [82,93].

Pooled procurement, the collective purchasing of medicines by multiple entities, allows for the negotiation of better prices [82]. Purchasing and promotion of generic medicines, which are significantly cheaper than patented medicines, is another method of ensuring affordable drugs [60]. The Pan American Health Organization (PAHO) Strategic Fund, the Organisation of Eastern Caribbean States (OECS) and the Joint Procurement Agreement (JPA) by the European Union are some examples of organizations that have successfully utilized a policy of pooled procurement in order to obtain a variety of therapies at a lower cost [95,96,97,98].

Many of these policies are repeatedly assessed against their ability to contain costs, but less in terms of their ability to provide access to medications [98].

The Patented Medicine Prices Review Board (PMPRB) is a Canadian regulatory authority that performs ongoing monitoring of patented medications. If there is concern regarding pricing which is deemed to be unreasonable, then public hearings may be held, following which orders may be issued to reduce a particular medication’s price [99]. Within the European Union, several countries employ strict and rigorous pricing models which have led to lower drug costs [93,94].

Collaborative approaches between regulatory authorities and pharmaceutical companies involving these pricing policy approaches and ensuring more transparency in terms of real medicine costs, research, and development will contribute towards the curtailment of high prices.

#### 5.1.2. Reimbursement Policies

Effective reimbursement policies ensure that out-of-pocket expenses are limited and that patients can afford their medications. Public insurance programs (such as Medicare and Medicaid in the US) can provide comprehensive public access to therapies which improve medication adherence and health outcomes.

Universal health coverage systems (such as those in Canada, the UK, and France) enable the public health system to cover most medications [100].

In countries with social health insurance systems, like Germany, mandatory health insurance covers most drug costs with modest copayments. Germany has an annual cap on out-of-pocket expenses which is set at 2% of household income, or 1% for the chronically ill [100,101]. Sweden implements a high-cost threshold system where patients pay the full price up to a certain amount, following which the government covers the costs [100].

While many healthcare systems contribute towards the cost of medicines, high copayments remain an economic barrier for many patients. Subsidies or reduced copayments based on an individual’s income can make medicines more affordable and improve access. Furthermore, providing exemption status for specific groups, such as children, the elderly, low-income individuals, or those with chronic conditions, can ensure that these groups have access to necessary medications [102,103,104].

Tiered formulary systems are often used by private insurers to manage the costs of drugs. Drugs are classified into different tiers based on efficacy and cost. For example, tier 1 would include generic drugs with no copayments. Tier 2 might comprise preferred brand-name drugs. Tier 3 would include high-cost specialty drugs where many novel lipid-lowering therapies may be found and carry higher copayments. While tiered formulary systems may improve access to affordable medications, the high out-of-pocket expenses for higher-tier medications remain a barrier for many patients [85,93].

Risk-sharing agreements are outcome-based contracts that tie reimbursement to the clinical efficacy of the therapy. This model has been applied by some insurers to PCSK9 inhibitors where reimbursement depends on specific reductions in LDL-C and cardiovascular events [68].

#### 5.1.3. Public Funding and Subsidies

Government-allocated funds could subsidize the cost of LLTs. Public health programs can provide these medications free to eligible patients, a system seen in various national health systems globally. However, although government subsidies for novel therapies may offset some costs, this strategy is likely unsustainable for many countries, particularly LMICs due to limited resources and competing healthcare priorities.

Public–private partnerships (PPPs) can be powerful means of leveraging both sectors. Joint ventures that focus on research and development of novel therapies allow for the pooling of resources and the sharing of both the risks and the benefits. The addition of public funding can be used to “de-risk” private investments in high-risk areas of drug development. Governments can offer grants, subsidies, or tax incentives to encourage private sector participation [105,106].

The establishment of prize funds and advanced market commitments to incentivize the development of novel therapies also can help address unmet medical needs. Furthermore, offering market exclusivity for pharmaceutical companies that develop and bring novel medications to market can incentivize development [105].

Intellectual property policies that strike a balance between rewarding innovation and ensuring access [107]. This can include measures like patent pools and promoting generic competition after patents expire. Encouraging voluntary licensing agreements where the patent holders grant licenses to manufacturers in LMICs to produce and distribute affordable versions of novel LLTs can improve access in underserved regions [105,106].

#### 5.1.4. Innovative Funding Models

The pharmaceutical industry has also recognized its own role in improving access to medications. The European Federation of Pharmaceutical Industries and Associations (EFPIA) has described the need for novel approaches to existing traditional pricing models [94]. Some of these approaches include outcome-based payments, whereby the payment for a medicine is dependent on its real-world performance and is based on measurable outcomes [94]. Over-time or staggered payments allow payers to make payments to manufacturers over a period while the patient receives therapy [94]. This mitigates the high upfront cost of medications. Subscription payments can be used to delink payment for a treatment from the number of patients who receive treatment [94].

Combination-based pricing addresses the notion that combination therapies are not necessarily the added value of medicines used separately [94].

### 5.2. Streamlining Regulatory Processes

While most initial claims for PCSK9 inhibitor therapy are rejected, engaging in an appeals process with insurers leads to positive outcomes in a significant number of cases [108].

The regulatory approval process can often be lengthy and complex, and streamlining this process can facilitate quicker patient access to novel therapeutics while still maintaining rigorous safety and efficacy standards.

Regulatory requirements can vary significantly between regions. Harmonizing these regulations can reduce redundancy, decrease approval times, and facilitate simultaneous launches in multiple markets.

Mutual recognition agreements (MRAs) allow regulatory agencies in different countries to rely on each other’s inspection reports and regulatory decisions. This reduces duplication and speeds up the approval process. At present, the FDA has MRAs with the EU, Switzerland, and the United Kingdom.

International regulatory bodies, such as the FDA (US) and EMA (Europe), work together through initiatives like the International Council for Harmonization of Technical Requirements for Pharmaceuticals for Human Use (ICH) [109]. The ICH brings together regulatory bodies and industry to facilitate discussion on the scientific and technical aspects of drug registration. Consistent guidelines on clinical trials, manufacturing standards, and drug evaluation can simplify the approval process across different jurisdictions [109].

Expedited approval pathways can significantly reduce the time required to bring novel LLTs to market, particularly for drugs that address unmet medical needs. This is particularly relevant for rare lipid disorders such as homozygous FH.

The FDA, as an example, has a number of expedited drug approval programs. Programs like the FDA’s Fast Track Designation aims to expedite the review of drugs intended to treat serious medical conditions. The Breakthrough Therapy Designation is granted by the FDA to drugs that show a marked improvement over existing therapies based on preliminary clinical evidence. The Accelerated Approval program allows for earlier approval of drugs that treat serious conditions and fill an unmet medical need [110]. Post-marketing studies are required to confirm the clinical benefits following accelerated approval [110].

Good post-market surveillance and pharmacovigilance allow regulatory bodies to monitor the safety and effectiveness of new therapies after their approval. This effectively provides a safety net that allows for earlier market access [111].

### 5.3. Public Health Interventions

#### 5.3.1. Telemedicine

The utilization of telemedicine allows patients living in remote areas to access medical care [112]. Telemedicine was initially established to assist with the timely management of acute conditions such as stroke, myocardial infarction, or trauma [113,114]. It has since expanded to encompass the comprehensive care of chronic diseases such as heart failure and diabetes [112,115,116]. As a result, telemedicine has become one of the fastest-growing healthcare delivery modalities in the US [112].

Access to healthcare can be considered in terms of three core components: entry into a health system, adequate supply of services, and timely provision of care [112]. Telemedicine can effectively address many limitations encountered by each of these aspects of healthcare access.

A significant barrier to entry into a health system is transportation. Cost, geographical distance, lack of reliable transportation, and inability to drive due to patient health status are major factors limiting patient access [117]. There is a growing shortage of physicians worldwide, particularly in rural areas and in LMICs [112,118]. Healthcare provider availability significantly influences access to treatment. Waiting times, which may be considerably longer for specialists, may also be a deterrent to accessing healthcare systems [112]. Telemedicine has been found to facilitate communication and increase collaboration between specialists and community healthcare providers.

Telemedicine has demonstrated similar, and sometimes better, health outcomes compared to the traditional models of care, and this is also true for improving access to LLTs [119,120]. There are, however, limitations to telemedicine, which may create their own barriers to access. These include the inability to perform clinical examinations, the loss of a patient–provider relationship, or the inability to respond to non-verbal cues during consultations [120]. Indeed, technological dexterity may vary across patient age groups, and the technological requirements for telemedicine may present an added cost to patients [120,121].

Interventions to improve access to telemedicine services in rural areas and LMICs might include improving technology infrastructure but also tailoring systems to patients and/or healthcare facilities with different technology infrastructure capabilities. The use of social media channels to improve awareness about telemedicine, providing dedicated outreach and technical support to people with limited access or familiarity with new technologies, and expanding insurance to include coverage for telehealth consultations are all effective strategies for improving access to telemedicine services that could be employed in rural areas and LMICs [122].

Disparities related to age, ethnicity, and socioeconomic status may, however, be exacerbated by telemedicine, creating further barriers to accessing treatment [112]. Confidentiality, legal liability, and data security are also legitimate concerns affecting a broader uptake of telemedicine [121].

Telemedicine has improved the geographical barriers to medical care and access, but to a lesser extent the social and economic disparities.

#### 5.3.2. Education and Advocacy

Addressing knowledge barriers requires multifaceted approaches. Healthcare provider behavior change is necessary [57]. Ongoing training and educational resources for healthcare providers can ensure they are equipped to prescribe novel therapies appropriately. For example, the PCSK9 Forum is a not-for-profit educational resource offered to healthcare providers at no cost [123].

Healthcare providers should also undergo training to recognize and address implicit biases, ensuring equitable treatment for all patients. Diversification of clinical trials too improves access for racial groups that are often under-represented [71]. However, addressing systemic racism requires multifaceted approaches at various levels, including policy reforms to promote health equity and anti-discrimination measures within healthcare systems [69].

For patients, forgetting to take medications is a significant barrier to adherence, particularly when polypharmacy is required to reduce ASCVD risk. Specific strategies like text reminders and fixed-dose combination (FDC) pills improve adherence [66]. Combining several CV medicines into one FDC form, or “polypill”, has been suggested to increase adherence, reduce delivery costs, and ease supply-chain burdens. A statin and ezetimibe FDC significantly improved LDL-C reduction and patient compliance compared to separate pills [124]. However, only 31.5% of patients using the FDC reached recommended LDL-C levels [124]. In a phase 3 trial, the FDC of bempedoic acid and ezetimibe outperformed ezetimibe alone and bempedoic acid alone [125]. These findings highlight the potential of FDCs to enhance LLT effectiveness and patient adherence.

Educational initiatives aimed at increasing patient awareness and improving public knowledge can also improve the uptake of novel therapies.

Patient advocacy groups and professional physician societies/organizations (such as the American Heart Association (AHA), European Society of Cardiology, National Lipid Association, and Family Heart Foundation) are important in raising awareness around lipid disorders and the need for access to effective LLTs. Indeed, a number of these groups were effective in campaigning for lower costs and broader access to PCSK9 inhibitors, which eventually led to a significant price drop in these therapies [126].

In order to overcome the barriers to access to innovation for patients who need it, a new initiative called SMASH is being deployed. The goal of SMASH (System and Molecular Approaches of Severe Hyperlipidemias) is to facilitate access to accurate diagnosis and precise treatments for patients with rare or severe lipid disorders, regardless of their economic status, mobility, or living environment, including patients in remote regions or emerging economies. SMASH has several dedicated platforms, accessible to the various actors and organizations involved in the world of rare or severe lipid diseases. This initiative is described in more detail elsewhere. To raise awareness about access to diagnosis and innovative treatments for patients with rare or severe diseases, individuals and organizations are strongly invited to sign the SMASH declaration (manifesto) at www.smash-access.org.

#### 5.3.3. Multidisciplinary Teams

Utilizing clinical services within the community pharmacy setting can also be used to reduce costs and improve access. Community pharmacists can assist with prior authorizations, the administration of drugs such as PCSK9 inhibitors, patient education, and follow-up point-of-care cholesterol testing [127].

Effective collaboration between primary care and specialist care teams can ensure that novel therapies are rolled out at the primary care level [30,127].

## 6. Future Directions and Recommendations

Collaboration between healthcare providers, policymakers, industry, and patient advocacy groups is important for addressing barriers to access.

Policymakers should look to implement pricing regulations and utilize innovative funding models that ensure affordability and access to LLTs for all patient populations. Streamlining regulatory processes will not only prevent delays in medication approvals but also ensure that quality standards are maintained. Investment within public health structures such as collaborative community-based programs, telemedicine, and educational initiatives for both patients and providers is recommended.

Healthcare providers should stay up to date on the latest guidelines and recommendations for the management of dyslipidemia, especially with regard to novel LLTs. Providers are important advocates for patient access to LLTs. Telemedicine and community-based services can reach underserved populations and improve medication adherence.

The pharmaceutical industry should consider novel pricing policies such as value-based pricing models and outcome-based payment arrangements which may make novel LLTs more affordable and accessible.

Patient advocacy groups should continue to advocate for policies and initiatives that promote equitable access to LLTs for all patients, regardless of socioeconomic status or geographical location; raise awareness about dyslipidemia and the importance of early detection and treatment to prevent cardiovascular events; and provide support and resources to patients navigating insurance coverage, medication affordability, and adherence to LLT regimens.

Acknowledging and addressing systemic racism as a barrier to accessing LLT will address the racial disparity that exists throughout medicine. Inclusive and diversified clinical trials that will be more representative of the population at large should be encouraged.

## 7. Conclusions

Statins will remain the cornerstone for lipid management for the foreseeable future. Improving access to statins and ezetimibe in LICs as a bare minimum would alleviate a major obstacle to care. However, when lifestyle interventions and statins are insufficient to meet LDL-C targets to reduce ASCVD, additional lipid-lowering therapies need to be considered. Novel lipid-lowering therapies are being developed rapidly, increasing the options to reduce the risk for ASCVD, but currently available drugs have been significantly underutilized worldwide.

While novel LLTs offer significant clinical benefits, their high costs, together with other barriers, limit their impact. Addressing these challenges through health policy interventions, insurance reforms, improved healthcare delivery, and education initiatives can improve access and ensure that more patients benefit from these advancements in dyslipidemia management.

## Figures and Tables

**Table 1:** Novel lipid-lowering therapies for LDL-C reduction.

Medication	Mechanism of Action	LDL-C Reduction	Phase of Development
Tafolecimab	Humanized mAb against PCSK9	~57–70%	Marketed
Lerodalcibep	PCSK9-binding domain (adnectin) conjugated with human albumin	~50%	Phase 3
Inclisiran	siRNA inhibition of hepatic PCSK9 synthesis	~50%	Marketed
MK-0616 (Enlicitide decanoate)	Oral PCSK9 inhibitor	~60%	Phase 3
CVI-LM001	Oral PCSK9 inhibitor	~26%	Phase 2
Cepadacursen	Long-acting ASO targeting PCSK9	Not known	Phase 2
Bempedoic acid	Inhibits ATP-citrate lyase	~24%	Marketed
Evinacumab	ANGPTL3 inhibitor	~50%	Marketed (for HoFH)
Zodasiran	siRNA targeting hepatic ANGPTL3	~20% (mixed dyslipidemia) ~48% (HoFH)	Phase 2
Solbinsiran	GalNAc-conjugated siRNA targeting hepatic ANGPTL3	36% reduction in ApoB	Phase 2b
Obicetrapib	Inhibits CETP	~45%	Phase 3
VXX-401	Anti-PCSK9 vaccine	~65% (NHPs)	Phase 1
VERVE-201	CRISPR/Cas9-based editing of *ANGPTL3*	~46% (NHPs)	Phase 1b
VERVE-101	CRISPR/Cas9-based editing of *PCSK9*	~46%	Phase 1
CTX310	CRISPR/Cas9-based editing of *ANGPTL3*	Not known	Phase 1

mAb = monoclonal antibody; PCSK9 = proprotein convertase subtilisin/kexin type 9; siRNA = small interfering RNA; ASO = antisense oligonucleotide; ANGPTL3 = angiopoietin-like protein 3; CETP = cholesterol ester transfer protein; HoFH = homozygous familial hypercholesterolemia; GalNAc = N-acetylgalactosamine; CRISPR = clustered regularly interspaced short palindromic repeats; NHP = non-human primate.

## Data Availability

Not applicable.
